# Optimizing the efficiency and implementation of cash transfers to improve adherence to antiretroviral therapy: study protocol for a cluster randomized controlled trial

**DOI:** 10.1186/s13063-020-04899-7

**Published:** 2020-11-23

**Authors:** Laura Packel, Prosper Njau, Carolyn Fahey, Angela Ramadhani, William H. Dow, Nicholas P. Jewell, Sandra McCoy

**Affiliations:** 1 School of Public Health, Division of Epidemiology, University of California, Berkeley, 2121 Berkeley Way, 5th Floor, Berkeley, CA 94720 USA; 2 Strategic Information and Research Unit, National AIDS Control Program (NACP), Ministry of Health, Community Development, Gender, Elderly, and Children, Dar es Salaam, Tanzania; 3 National AIDS Control Programme (NACP), Ministry of Health, Community Development, Gender, Elderly, and Children, Dar es Salaam, Tanzania; 4 School of Public Health, Division of Health Policy and Management, University of California, Berkeley, 2121 Berkeley Way, 5th Floor, Berkeley, CA 94720 USA; 5 Department of Medical Statistics, London School of Hygiene & Tropical Medicine, Keppel Street, Bloomsbury, London, WC1E 7HT UK; 6 School of Public Health Biostatistics Division & Department of Statistics, University of California, Berkeley, 2121 Berkeley Way, 5th Floor, Berkeley, CA 94720 USA

**Keywords:** HIV, Tanzania, Implementation–effectiveness hybrid design, Cash transfer, ART adherence, Cluster randomized trial

## Abstract

**Background:**

Antiretroviral therapy (ART) for HIV, taken daily, is an effective strategy to clinically suppress the virus, providing the dual benefit of improved survival and vastly decreasing the risk of transmission. However, this highly effective intervention has not yet reached all who could benefit. Cash transfers are increasingly recognized as an effective strategy to motivate behavior change and improve HIV care and treatment outcomes, including engagement in HIV care and adherence to ART. Despite a growing evidence base and strong theoretical foundation for the cash transfer approach, key questions remain. To address these questions and begin to bridge the “know-do gap” with respect to cash transfers, our team is employing an implementation science approach to iterative development of an incentive-based intervention to promote ART uptake and adherence among people living with HIV (PLHIV) in the Lake Zone region, Tanzania.

**Methods:**

We will conduct a type I hybrid implementation–effectiveness trial to test the effectiveness of a cash transfer intervention on the outcome of HIV viral suppression, and concurrently examine the potential for real-world implementation with a mobile health technology (mHealth) system. Specifically, our team will expand the intervention to 32 clinics and enroll 1984 PLHIV to (a) evaluate its effectiveness by conducting a cluster randomized controlled trial with clinics as the unit of randomization and 12-month viral suppression as the primary outcome and (b) evaluate the implementation challenges and successes at multiple levels (patient, provider, clinic).

**Discussion:**

This trial will provide evidence not only about the real-world effectiveness of cash transfers for retention in HIV care and viral suppression, but also on the implementation challenges and successes that will facilitate or hinder wider scale-up within Tanzania and beyond.

**Trial registration:**

ClinicalTrials.gov NCT04201353. Registered on December 17, 2019

## Contributions to the literature

- Adherence to HIV antiretroviral therapy and resulting viral suppression are widely known as critical for ending the HIV epidemic.
- Effective implementation strategies to ensure that people living with HIV have viral suppression are needed; cash transfers are one such strategy.
- This study protocol describes a cluster randomized controlled trial with an implementation–effectiveness hybrid design to optimize a cash transfer implementation strategy for HIV control in real-world settings.

## Background

Antiretroviral therapy (ART), taken every day as prescribed, is an effective strategy to clinically suppress the HIV virus, providing the dual benefit of improved health and vastly decreasing or eliminating the risk of onward transmission. Despite the robust evidence, this highly effective intervention has not yet reached all who could benefit. For example, in Tanzania, 1.6 million people are living with HIV, and 72,000 new infections occur yearly. Of those living with HIV, only 62% are virally suppressed, and of those who are currently on HIV treatment and who have viral load testing results, 87% are virally suppressed [1, 2]—falling short of the 95–95–95 goals that UNAIDS has set for 2030 for which 95% of those on HIV treatment should be virally suppressed [2]. To reach these goals, new and effective strategies are needed that can translate evidence into widespread practice and thereby bring sustained HIV treatment for viral suppression to all people living with HIV (PLHIV).

Conditional economic incentives are increasingly recognized as one such implementation strategy: these programs typically provide cash (or other incentives) to people who meet a particular condition, for example testing for HIV, returning for HIV test results, or testing negative for sexually transmitted infections, thus motivating certain behaviors that result in improved health. Conditional economic incentive programs evaluated in the context of improving HIV outcomes have largely been short term and have primarily been implemented in the context of research settings, differentiating them from social protection cash transfer programs that are typically longer term and funded by the government. There is now a substantial evidence base demonstrating the effectiveness of cash transfer programs in improving outcomes along the HIV care continuum in low-resource settings in a research context [3–23]. Despite this strong evidence, few of these cash transfer programs to improve HIV outcomes have been scaled, and gaps exist in understanding the long-term impacts of these programs.

To address these questions, we are employing an implementation science approach to evaluate a cash incentive program designed to promote ART adherence among PLHIV in the Lake Zone, Tanzania. Over the past several years, our team has designed and conducted a set of iterative experiments to determine the *efficacy* of the incentive-based intervention, *optimize* the intervention for a real-world clinical setting, and assess the feasibility and acceptability of the intervention package for scale-up and sustainability. The effectiveness trial described in this protocol is the logical next step in evaluating incentive-based approaches for improved HIV care outcomes. Together, this set of trials (efficacy–optimization–effectiveness) will generate an evidence base for the most effective, incentive-based implementation strategy for the clinically proven intervention of ART adherence.

Results from the first two randomized trials conducted by our team (efficacy and optimization) demonstrated that cash transfers conditional on visit attendance have the potential to improve ART adherence and retention in care among PLHIV in Tanzania. In the first study (efficacy), our team randomized 800 food-insecure PLHIV who recently started ART at three clinics to the standard of care or 6 months of cash or food transfers, conditional on visit attendance [24]. After 6 months of the intervention, we found that short-term cash transfers were superior to the standard of care on all indicators of adherence and retention, including the medication possession ratio (MPR), a pharmacy-based measure of adherence associated with viral suppression [25–28], appointment attendance, and loss to follow-up [20]. At 12 months, 6 months *after* the *intervention ended*, the cash group remained more likely to be in care than the standard of care group and had superior appointment attendance. Furthermore, cash transfers were safe to administer [29, 30] and, compared to food baskets, were equal or superior to food support on all outcomes, were cheaper and easier to monitor, and were preferred by patients. Analysis of individual motivation found that cash transfers do not undermine intrinsic motivation to adhere to HIV treatment; indeed, intrinsic motivation *increased* over time as PLHIV experienced the benefits of treatment [31]. Qualitative research revealed that money received as part of the intervention was largely being spent on food, school fees, and investment in assets and small businesses.

Building on these proof-of-concept results, a second trial (optimization) evaluated the cash transfer intervention among 530 PLHIV initiating ART at four clinics. This second study (completed in August 2019, ClinicalTrials.gov: NCT03351556) evaluated two cash transfer sizes (~ $5 and ~ $10) conditional on visit attendance and, for the larger amount, confirmed the first trial’s results using viral suppression as the outcome at 6 months (risk difference vs. control = 13.0 percentage points, 95% CI 4.5–21.5) [19]. The study also assessed whether a clinic-based mobile health technology (mHealth) system could streamline intervention implementation; the system was designed to identify patients biometrically (using fingerprints) and to automatically transfer cash via mobile money to eligible patients upon scanning in for their visit [32].

Armed with evidence on the intervention’s efficacy and an optimized implementation strategy using the larger cash amount and mHealth system, we will now conduct the third study, a type I hybrid implementation–effectiveness trial [33]. This study will test the effectiveness of the cash transfer intervention on the outcome of viral suppression 6 months after the cash program ends and concurrently examine the potential for real-world implementation through measurement of implementation science constructs [33]. Specifically, our team will expand the intervention to 32 clinics to (a) evaluate its effectiveness by conducting a cluster randomized controlled trial with clinics as the unit of randomization and 12-month viral suppression as the primary outcome—a key indicator used in monitoring global progress towards HIV epidemic control; and (b) evaluate the challenges and successes by measuring implementation science outcomes at multiple levels (patient, provider, clinic) following Proctor’s framework and constructs [34]. This is the next, logical stage of this research as we build the evidence base for the eventual adoption of a streamlined version of this intervention at scale.

## Methods

### Design

The study has two primary objectives:

1. *Impact evaluation: Evaluate the effect of the 6-month cash transfer program on viral suppression (< 1000 copies/ml) at 12 months after starting ART.*
  The primary objective of this study is to evaluate the effectiveness of the cash transfer program using 22,500 Tanzanian Shillings (TZS)/month (as determined by phase I) at improving the proportion of PLHIV retained on ART and with suppressed viral load at 12 months after starting ART. Using a cluster randomized control trial, with our sample size of 32 clinics and 1984 participants (62 participants per clinic), we will have 80% power to detect a risk difference (RD) of 11 percentage points in viral suppression at 12 months between the participants attending intervention and control clinics.
  The secondary outcomes of the impact evaluation are:
  Viral suppression (< 1000 copies/ml) at 6 months;
  Retention on ART at 6 and 12 months;
  The proportion virally suppressed of those retained on ART at 6 and 12 months; and
  Appointment attendance, the proportion of scheduled visits that were completed during the 0–6- and 0–12-month periods.
2. *Implementation study: Understand implementation successes and challenges through measurement of implementation outcomes, and use lessons learned to inform wider adoption of cash transfer programs for PLHIV.*
  Although evidence from our previous studies demonstrates the preliminary effectiveness of cash transfers on HIV-related outcomes, we do not yet know the optimal strategy for implementing this type of system at a larger scale and outside of a research setting. Thus, in anticipation of the potential adoption of the program by the Ministry of Health or others, a primary objective of this study is to gather information related to successful implementation practices and challenges needing attention before scaling.

**Fig. 1:**
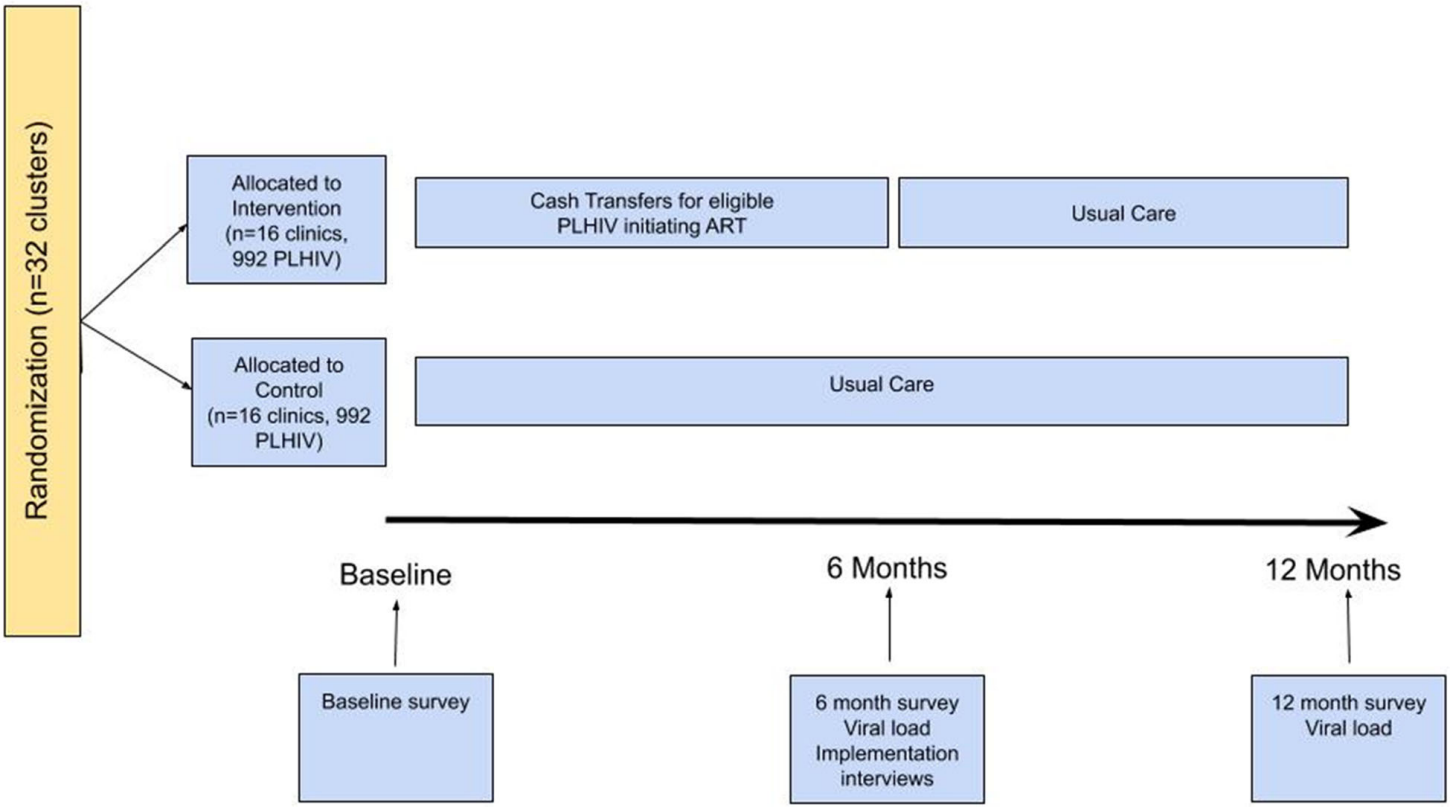
Cluster randomized hybrid effectiveness–implementation trial study design

To address these aims, we will conduct a type I hybrid implementation–effectiveness study using a two-arm, cluster randomized controlled trial (objective #1, “impact evaluation”) with the health facility as the unit of randomization (see Fig. 1). To measure implementation outcomes (objective #2, “implementation study”) following Proctor’s framework [34], quantitative and qualitative interviews will be conducted with clinic staff (pharmacy staff, registration staff, clinicians), clinic management, and patients at participating sites. Results will be used to assess heterogeneity in implementation practices and thus inform optimization for potential scale-up. The study will be conducted at 32 HIV primary care clinics in the following Lake Zone regions in Tanzania: Shinyanga, Mwanza, Kagera, and Geita.

### Theoretical framework

The use of incentives for ART treatment adherence is supported by several theories, including Self-Determination Theory, which describes engagement in an activity because of an external reward like a cash transfer [35]. In addition, microeconomic theory posits that people acquire more of a less costly good and less of a more expensive one [36] and that individuals often have high discount rates or “present-biased preferences,” placing disproportionate weight on the present while largely ignoring the future [37, 38]. An implication is that when a behavior, like attending an HIV care visit, has small immediate costs and large delayed benefits, a small immediate incentive may counteract the present costs and tip the balance towards the positive behavior [7, 37]. The use of incentives for behavior change is also supported by behavioral economic theory, which incorporates constructs from psychology to account for the predicable irrationalities, heuristics, and biases of human behavior [39]. For example, “nudges” or short-term, small incentives can change behavior [40, 41] and create new habits [42]—a goal of the short-term cash transfer intervention.

The implementation science portion of the hybrid trial is guided largely by Proctor’s implementation science framework, which explores implementation successes and challenges at multiple levels, including the patient, provider, and clinic [34]. The study design follows closely the typology of hybrid designs outlined by Curran et al. [33] (type I in our case), for which the primary question is related to whether the intervention will work in real-world clinical settings and the secondary question relates to the potential barriers and facilitators of widespread adoption of the intervention (Proctor’s framework).

### Clinic recruitment

We will engage Regional Medical Officers (RMOs) in Shinyanga, Geita, Mwanza, and Kagera Regions, Tanzania, to create a sampling frame of eligible study facilities within each region. Initial eligibility criteria for clinics are as follows:

- Currently use an electronic medical record database;
- Average of at least 65 new ART initiates per quarter in 2019, with no fewer than 35 ART initiates in any single quarter in 2019;
- Within 100 km driving distance of a city center (either Bukoba, Mwanza, Geita, Kahama, or Shinyanga cities); and
- A minimum of 15 km from another study clinic.

The team will approach and enroll eligible selected sites in a randomly assigned order (1 to 10 within each region) until the target enrollment is reached, with the goal of having 32 sites enroll in the study.

### Study arms

#### Control arm

Participants in the control arm will receive the standard of care for PLHIV per the National Guidelines in Tanzania [43], as delivered by the participating health facilities. In addition to the standard of care, the mHealth system developed and utilized in the previous (optimization) trial will be implemented in all study clinics (intervention and control). This system was successfully used in the clinics to enroll study participants, track patient appointment attendance, and disburse cash transfer payments for those eligible using automated mobile money transfers [32]. We will adapt this mHealth system for the current study and implement it in *all study facilities* (intervention and control) for enrolling participants, tracking participant visits, sending appointment reminders, and sending cash transfers via mobile banking (cash transfer group only; consistent with procedures used in our previous studies). This strategy is consistent with the Ministry of Health goal of using mHealth strategies for patient care management in Tanzania, as incorporated into the National Strategy for HIV Care [44].

#### Intervention arm

In addition to the mHealth system as described above, participants in the intervention arm will have the opportunity to receive up to 6 consecutive monthly cash transfers of 22,500 TSH (~ $10) each, conditional on visit attendance with the HIV care provider. Cash transfers will be given once monthly for *up to* 6 months, spaced ≥ 25 days apart (consistent with National Guidelines for monthly or bimonthly visits [43]). This means that the cash transfer is only given when the patient visits the clinic for their routine appointment, regardless of whether the visit is earlier or later than the scheduled appointment (but no earlier than 25 days since the last transfer). Upon the monthly visits when a patient checks in for their appointment (via fingerprint or entry of clinic ID), the cash transfer will be automatically distributed through the mHealth system. Transfer amounts are exclusive of transaction fees (< $1), which will also be included in the transfer. Thus, the intended amount is transferred to the patient upon withdrawal.

If a participant does not have a mobile money account, (1) s/he can sign up and return to the clinic on a different day to link this account to the mHealth system or (2) s/he may ask a trusted friend or relative to receive the transfer. Mobile money is a secure and convenient way for financial transactions, as mobile money kiosks are ubiquitous in the study regions and mobile phone ownership in Tanzania is high and approaching levels in the USA (75% in 2018 and rapidly increasing [45]). For participants who do not have mobile banking, wherever possible, we will make referrals to kiosks where they can sign up for the service, if desired. In the most recent study, we found that 88% of participants had access to a mobile phone and 79% had access to mobile banking.

#### Rationale for the cash amount and duration

Our now-completed study to optimize the intervention found that both cash transfer amounts (10,000 TSH and 22,500 TSH per month) significantly improved 6-month viral suppression versus the standard of care. At 6 months, we found a positive trend between incentive size and HIV viral suppression (OR = 1.10 per 2500 TZS, 95% CI 1.03 to 1.17). Further, we found that both cash amounts significantly improved HIV viral suppression over the comparison group [10,000 TSH 83% vs. 73%, risk difference (RD) = 10.2, 95% CI 1.6–18.8; 22,500 TSH 85% vs. 73%, RD = 12.1, 95% CI 3.6–20.6]; there was no significant difference between incentive groups (RD = 1.9, 95% CI − 5.8 to 9.7). However, only the larger cash value yielded significant improvements over the standard of care for the secondary outcomes of retention in care (RD = 7.1, 95% CI 0.3 to 13.9) and viral suppression among the subset retained in care (RD = 7.8, 95% CI 0.9 to 14.7). To determine the final cash transfer size for the current study, we followed our pre-registered analysis plan (at AsPredicted), as well as several additional analyses including a cost analysis; discussions with local stakeholders, including the Regional Medical Officers, and co-investigators; assessment of budgetary trade-offs associated with a smaller cash transfer versus larger study size; and consideration about the benefits and weaknesses of various cash amounts (including power considerations and the ethics of engaging in a statistically underpowered study; for example, powering the study for the smaller amount would require twice as many clinics in the study, or the study would be significantly underpowered). In addition, with expansion to additional regions, some with higher average levels of socioeconomic status than the region included in the optimization trial, there was some concern that the smaller transfer would be of lower value and thus not enough to improve adherence. Finally, the larger cash amount is of comparable magnitude to the monthly payments distributed through Tanzania’s social action fund, TASAF. After considering these factors, many of which are context-specific, we determined that the effectiveness evaluation will include a cash transfer amount of 22,500 TSH per month for 6 months.

The intervention is intended to fit within the differentiated model of care in Tanzania, in which patients starting ART are scheduled monthly clinic visits until the first viral load test at 6 months after ART initiation. At that time, “stable” patients (those who have attained viral suppression) are given 2-/3-month refill appointments and/or the opportunity to send a substitute for ART refills or to obtain refills at community-based locations. However, at 6 months, unstable patients (those without viral suppression) continue monthly visits, with enhanced adherence interventions or change of regimen, depending on clinical, immunological, and virological criteria, until they are determined to have stabilized. Note that we will track participation in enhanced adherence counseling among participants in both arms and present this in our results. Thus, the intervention is intended to support patients during the vulnerable first 6 months of treatment before the 6-month decision point for access to a less intensive clinic schedule.

### Randomization and masking

Once facilities are selected for the study and have agreed to participate, we will randomize 32 facilities to either the SOC or the cash transfer intervention group at UC Berkeley using the cvcrand package (cvrall command) in R statistical software [46]. To ensure that the arms are balanced on important covariates, and to mitigate the possibility of ineffective randomization due to the small numbers of clinics in the study, we will use a constrained randomization process [47]. This process essentially pre-determines a set of acceptable allocations into intervention and control clusters based on covariates of relevance and then randomly selects an allocation from the list of acceptable allocations [47]. Specifically, based on our experience in phase 1, we will include the following covariates in the constrained randomization process: geographic region (Geita, Kagera, Mwanza, or Shinyanga), facility level (hospital, health center, or dispensary), driving distance to a major city (km), proximity to a major road (< 5 km), and average number of ART initiates per quarter. We will stratify on geographic region. The 32 sites will be randomized 100,000 times, and we will select the unique schemes as the randomization space. Those iterations with an l2 balance score less than the *q* = 0.1 cutoff will be retained. Among those remaining iterations where there was little to no imbalance detected, we will check for validity of the constrained randomization (e.g., no deterministic allocation of clusters into arms) and ensure that there are sufficient constrained randomizations from which to randomly select a single randomization scheme.

Facility staff will not be blinded to intervention assignment. However, other clinic staff will not be informed that there are intervention and control clinics in the study, and clinical staff trainings for intervention clinics and control clinics will be conducted separately. In addition, participants will not be told during the consent process that as part of the study there are intervention and control clinics. The rationale for this is to prevent behavioral changes such as patients transferring from control to intervention clinics if they find out that there are some clinics offering cash transfers to new ART clients. Although this is not expected to be a significant problem based on feedback from local and regional health authorities, given the small incentive value and that participating facilities will be at least 15 km apart, large numbers of transferring patients could compromise the integrity of the study and will create an undue burden for facility staff at intervention clinics.

### Recruitment of participants

#### Impact evaluation

Recruitment *of individuals* for participation in the impact portion of the study will take place within the enrolled clinics. All patients at study clinics who meet the following inclusion criteria will be offered the opportunity to participate in the study:

- Greater than or equal to 18 years of age
- Living with HIV infection
- Initiated on ART (for the first time) less than or equal to 30 days prior to enrollment in the study
- Have access to a mobile phone (ownership, shared ownership, or access to a trusted person’s phone)
- Do not intend to transfer to a different facility for HIV care within the next 12 months

Eligible PLHIV at both intervention and standard of care sites will be automatically identified by the mHealth system upon registration in the system at a routine visit. An automatic prompt will direct clinic registration staff (who will manage the process) to the appropriate consents and forms in the mHealth system, including permission to access patient data as part of the study. If the patient consents, s/he will automatically be assigned a study ID. The study will use non-competitive enrollment so that we can achieve approximately equal numbers of study participants per site, with a goal of 62 per site. Sites that reach this goal early will discontinue enrollment.

#### Implementation study

Recruitment for participation in the implementation science portion of the study for clinic staff will take place in the intervention clinics once all participants have reached their 6-month post-ART timepoint and the cash transfer period (of 6 months) has ended. We will purposefully select staff from all study clinics such that our interviews include all levels of staff impacted by the changes in the clinic as related to implementation of the intervention (e.g., nurses, registration staff, physicians, In-Charges, pharmacists). Similarly, patients who have consented to and enrolled in the study will be purposefully sampled to assess their experiences with the cash transfer program and the mHealth system after their completion of cash transfer eligibility at 6 months. Specifically, we will interview patients who (a) received all six cash transfers, (b) received three or fewer of the six possible transfers, (c) enrolled in mobile money as a result of the study, (d) were virally suppressed at 6 months, and (e) were not virally suppressed at 6 months in addition to ensuring representation of both men and women and a range of ages.

### Outcomes and data collection

#### Primary outcome measure (impact evaluation, objective #1)

The primary endpoint is viral suppression at 12 months, defined as the proportion of PLHIV retained in HIV primary care and with suppressed HIV viral load 12 months after starting ART. The primary outcome is expressed as a binary variable, defined as PLHIV who are on ART and with sufficient HIV viral suppression (< 1000 copies/ml, WHO’s threshold for virologic failure in low- and middle-income countries [48]) versus not on ART or viral failure (≥ 1000 copies/ml). This outcome definition reflects global “treatment as prevention” strategies including the UNAIDS 95–95–95 targets, which aim for at least 95% of PLHIV to be on ART and 95% of those on ART virally suppressed. Patients considered not on ART include those who died, stopped ART and/or disengaged from care, or have not apparently received ARVs for ≥ 28 days since their last missed pharmacy pick-up [i.e., are lost to follow-up (LTFU)] following PEPFAR Monitoring, Evaluation, and Reporting Indicator Reference Guide Version 2.3 for current ART [49].

#### Secondary outcome measures (impact evaluation, objective #1)

- Viral suppression (< 1000 copies/ml) at 6 months;
- Retention on ART at 6 and 12 months;
- The proportion virally suppressed of those retained on ART at 6 and 12 months; and
- Appointment attendance, the proportion of scheduled visits that were completed during the 0–6- and 0–12-month periods.

#### Primary outcome measures (implementation study, objective #2)

Implementation outcomes assessed through in-depth interviews and surveys with patients and clinical staff: adoption, acceptability, penetration, fidelity, feasibility, appropriateness, and sustainability, plus usability (Table 1).

**Table 1:** Implementation study: data collection approaches and Proctor’s implementation science constructs

Data collection approach	Implementation outcomes	Outcome indicators
Patient survey (baseline, 6 months)	Adoption (mobile money)	Initiation of use of mobile money; satisfaction with the program generally and the mHealth system specifically
	Acceptability	
Clinical staff survey (6 months)	Acceptability	Staff support and acceptance, level of institutionalization
	Penetration	
	Fidelity	
	Feasibility	
In-depth interviews with clinic staff (6 months)	Acceptability	Staff support and acceptance, staff burden, practicality, and perceived fit
	Appropriateness	
	Sustainability	
In-depth interviews with patients plus usability survey (6 months)	Acceptability	Patient satisfaction with program and delivery model, impact on patient, practicality and perceived fit, adoption of mobile money
	Appropriateness	
	Adoption	

#### Data collection

Data collection will include the following:

- Medical record review for the primary outcome, viral suppression, at 6 and 12 months
- Surveys with patients at intervention and control clinics at baseline and 6 and 12 months (*n* = 1984)
- In-depth interviews with patients in the intervention clinics at 6 months (*n* = 30)
- Surveys with clinical staff at intervention and control clinics at 6 months (*n* = 110)
- In-depth interviews with clinical staff at intervention and control clinics at 6 months (*n* = 40)

We linked each data collection activity for the implementation study to implementation outcomes in the Proctor et al. framework (Table 1) [34]. We will analyze data from structured surveys and in-depth interviews to examine the implementation constructs of acceptability, penetration, sustainability, appropriateness, adoption, feasibility, and fidelity. We will use validated surveys to assess the usability of the mHealth system from both the clinic staff and patient perspectives, including the System Usability Scale [50] and the Health Information Technology Usability Evaluation Scale [51].

#### Participant tracing—impact evaluation

As retention in care is included as part of the definition of the primary outcome of this study (i.e., those not retained on ART are included in the denominator of the primary outcome of viral suppression), no additional retention activities will be implemented other than the routine procedures already in place and led by clinical staff. According to national guidelines, patients who miss a scheduled appointment are tracked in the community by a system of home-based care providers (HBCs); this system will remain in place during the study. In addition, we will closely track implementation of patient tracking as specified in the national guidelines within each study clinic so that we can capture any variation in implementation at the clinic level. Research staff will not be involved in tracing participants at any study facility during the 12-month follow-up of each participant. At endline only (12 months), research assistants will enhance these procedures with additional robust tracing using “gold-standard” tracing methods (at least 3 tracing attempts using multiple methods), procedures to investigate all potentially LTFU patients, confirm “silent transfers” (those who transfer to new facilities without notification of the prior facility) and deaths, and refer patients to health facilities where missing plasma specimens can be collected by health facility staff for viral load quantification.

### Statistical analysis

#### Power calculation

The sample size was determined for the impact study to evaluate the effectiveness of the cash transfer program at improving the proportion of PLHIV with suppressed viral load (< 1000 copies/ml) at 12 months. We used estimates of viral suppression at 12 months and ICC = 0.05, estimated from a previous cluster randomized study of HIV treatment adherence that we conducted among HIV-infected pregnant women in Shinyanga Region, an ICC consistent with previous studies [52]. Using site-adjusted data about viral suppression at 6 months from phase I (75.2% in the control group), along with estimates of viral suppression decline between 6 and 12 months, we estimate that viral suppression among adults at 12 months in the comparison sites will (conservatively) be 70%. With an ICC of 0.05, 32 facilities, 80% power, and 56 PLHIV per clinic (*n* = 1792), the minimum detectable effect is 11 percentage points as an absolute reduction in the proportion experiencing viral suppression. In the second trial (optimization), we achieved a 12.6 percentage point difference with the 22,500 TSH cash transfer at 6 months, so we believe this effect size is achievable. In addition, if viral suppression is lower than expected in the comparison arm, and the effect of the intervention remains stable, power will increase. We will inflate the target sample size by 10% to *n* = 1984 (62 participants per clinic across 32 clinics) to account for unexpected issues or significant differences in the enrollment between clinics.

#### Data analysis—impact study

We will conduct an intent-to-treat analysis to determine the effect of the intervention on 12-month viral suppression (the primary outcome). We will conduct a cluster-based permutation test on the individual-level outcome data, which accounts for clustering within the clinic. The effect estimate of interest is the risk difference of viral suppression among PLHIV attending intervention versus comparison facilities. We will pre-register our analysis plan at AsPredicted.

We will conduct several secondary analyses of the primary outcome, although we are not powered for these analyses. We will examine heterogeneity in the primary analysis by facility and patient characteristics. We will also conduct a treatment-on-the-treated analysis to isolate the impact of incentive delivery [53–55].

Using the same methods as for the primary analysis, we will also assess effects on the following key secondary outcomes:

- Viral suppression (< 1000 copies/ml) at 6 months;
- Retention on ART at 6 and 12 months;
- The proportion virally suppressed of those retained on ART at 6 and 12 months; and
- Appointment attendance, the proportion of scheduled visits that were completed during the 0–6- and 0–12-month periods.

We will additionally assess effects on the following survey-based outcomes at 6 and 12 months:

- Food security
- Mental health (anxiety, depression)
- Hopefulness
- IPV
- Self-efficacy
- Participation in the labor force/functional status
- Other indicators of household welfare (e.g., investment in small businesses)

#### Data analysis—implementation study

Following each in-depth interview, the interviewer will memo then debrief with the study team to discuss emergent themes. Debriefings will be held weekly with interviewers and the PI to ensure consistency and quality of data collection. Audio-recordings from IDIs will be verbatim transcribed and translated into English. Data analysis will follow an open-coding approach [56, 57] and will be based on research questions, study aims, and Proctor’s implementation constructs. An initial coding framework will include a list of predefined analytical terms relating to Proctor’s constructs; however, data analysis will be iterative, allowing new themes to emerge throughout the analysis process [58]. Data will be coded independently by two members of the research team using Dedoose qualitative coding software. Concepts will be grouped into categories, and main themes will be extracted and summarized in an analytic theme matrix.

Implementation outcome data from structured surveys with clinical staff will be analyzed descriptively using both STATA and Dedoose. We will synthesize quantitative and qualitative data to produce a comprehensive set of implementation outcome indicators. We will then use the implementation indicators to assess variation in how the program was implemented across the intervention clinics, for example, exploring levels of penetration and fidelity of the mHealth system, and explore variation in effectiveness using a sensitivity analysis. Note that we are not powered to show statistical differences in viral suppression by variation in implementation outcomes.

## Discussion

Achieving global goals for HIV epidemic control will necessitate new implementation strategies to identify PLHIV, link them to high-quality HIV care and treatment, and retain them on lifelong ART. Cash transfers have emerged as a promising strategy worthy of consideration as part of a comprehensive approach to HIV prevention and care. We have used an iterative process grounded in implementation science approaches to demonstrate the efficacy of cash transfers for ART adherence (trial 1) [20] and optimize the cash intervention with respect to cash amount (trial 2) [19]. Now, in our third trial, we will evaluate the effectiveness of cash transfers on viral suppression with a contemporaneous assessment of implementation outcomes with the goal of preparing the intervention for scale in the complex context of low- and middle-income country health systems. The results from this type I hybrid implementation–effectiveness trial will provide much-needed data on not only the real-world effectiveness of cash transfers for ART adherence and viral suppression, but also the implementation challenges and successes that will facilitate or hinder wider scale-up within Tanzania and beyond.

The hybrid implementation science/effectiveness approach will permit (a) a contribution to what is known about the use of short-term cash transfers for PLHIV in a real-world setting in sub-Saharan Africa, including their *long-term effects on viral suppression*, and (b) an understanding of how to optimize implementation approaches, and thus, provide guidance for policymakers in facilitating successful scale-up. These results have the potential to provide the evidence needed to show that short-term assistance confers lasting benefits for those starting treatment, providing needed support as they begin to feel the beneficial effects of ART. Furthermore, the study is based in the Lake Zone region of Tanzania, a region that faces challenges common throughout sub-Saharan Africa: a shortage of skilled personnel, poverty and food insecurity, and pervasive challenges with retention in care. For these reasons, our results are likely to have a high level of external validity and policy relevance in sub-Saharan Africa.

We have intentionally designed the hybrid trial to be largely self-sufficient—that is, clinical staff have the majority of interaction with the research participants, with support from research staff initially, and then only as needed. This approach comes with some trade-offs and limitations. First, there is the strong possibility that implementation of the intervention may differ by site. While implementation differences have the potential to dilute the effect of the cash program, for an effectiveness trial in a real-world setting, such variation will provide essential knowledge that will allow best practices and lessons learned to emerge and guide policymakers in bringing the program to scale. Second, patients attending control clinics may hear about the intervention and transfer out of control clinics and into intervention clinics. To mitigate this possibility (though this was not an issue in the first two trials), we have included a minimum geographic distance between clinics to minimize spillover effects and prevent contamination of comparison communities. In addition, as large numbers of transferring patients would compromise the integrity of the study and will create an undue burden for facility staff at intervention clinics, we will not reveal that there are intervention clinics where patients will receive cash transfers as part of the study to patients enrolling in the study at control clinics. Third, there is substantial variability over time and across clinics in the number of new ART initiates, and this variability may potentially impact the rate at which we are able to enroll patients into the study.

The results of this hybrid implementation–effectiveness trial will guide policymakers in Tanzania and elsewhere in the Region about whether this intervention should (or should not) be considered in national HIV/AIDS programs, and if it should, how implementation should proceed to optimize the effectiveness of the intervention in clinics across the country. Consequently, this effectiveness trial has been designed with an eye towards *real-world*, *future scale-up*, such that the procedures we propose to follow align as closely as possible with the National policy and consistent with what clinics would and could do outside of a research study setting.

## Trial status

Protocol version: 1.2

Recruitment start date: pending, recruitment scheduled to begin in November 2020

Anticipated recruitment completion date: May 2021

## Data Availability

Not applicable

## Abbreviations

ART: Antiretroviral therapy
PLHIV: People living with HIV/AIDS
TSH: Tanzanian Shillings
LTFU: Loss to follow-up
RA: Research assistant
CTC: Care and treatment clinic (HIV)
IS: Implementation science
TASAF: Tanzania Social Action Fund
RMO: Regional Medical Officer
HBCs: Home-based care providers
